# Effect of Tactile Sensory Substitution on the Proprioceptive Error Map of the Arm

**DOI:** 10.3389/fnins.2021.586740

**Published:** 2021-07-06

**Authors:** Justin Tanner, Gerrit Orthlieb, David Shumate, Stephen Helms Tillery

**Affiliations:** ^1^School of Biological and Health Systems Engineering, Arizona State University, Tempe, AZ, United States; ^2^Stanford School of Medicine, Stanford University, Stanford, CA, United States

**Keywords:** proprioception, multisensory integration, vibrotactile, electotactile, sensory substitution

## Abstract

Proprioceptive error of estimated fingertip position in two-dimensional space is reduced with the addition of tactile stimulation to the fingertip. This tactile input does not disrupt the subjects’ estimation strategy, as the individual error vector maps maintain their overall geometric structure. This relationship suggests an integration of proprioception and tactile sensory information to enhance proprioceptive estimation. To better understand this multisensory integration, we explored the effect of electrotactile and vibrotactile stimulation to the fingertips in place of actual contact, thus limiting interaction forces. This allowed us to discern any proprioceptive estimation improvement that arose from purely tactile stimulation. Ten right-handed and ten left-handed subjects performed a simple right-handed proprioceptive estimation task under four tactile feedback conditions: hover, touch, electrotactile, and vibrotactile. Target sets were generated for each subject, persisted across all feedback modalities, and targets were presented in randomized orders. Error maps across the workspace were generated using polynomial models of the subjects’ responses. Error maps did not change shape between conditions for any right-handed subjects and changed for a single condition for two left-handed subjects. Non-parametric statistical analysis of the error magnitude shows that both modes of sensory substitution significantly reduce error for right-handed subjects, but not to the level of actual touch. Left-handed subjects demonstrated increased error for all feedback conditions compared to hover. Compared to right-handed subjects, left-handed subjects demonstrated more error in each condition except the hover condition. This is consistent with the hypothesis that the non-dominant hand is specialized for position control, while the dominant is specialized for velocity. Notably, our results suggest that non-dominant hand estimation strategies are hindered by stimuli to the fingertip. We conclude that electrotactile and vibrotactile sensory substitution only succeed in multisensory integration when applied to the dominant hand. These feedback modalities do not disrupt established dominate hand proprioceptive error maps, and existing strategies adapt to the novel input and minimize error. Since actual touch provides the best error reduction, sensory substitution lacks some unidentified beneficial information, such as familiarity or natural sensation. This missing component could also be what confounds subjects using their non-dominant hand for positional tasks.

## Introduction

Cutaneous and deep sensations have some surprising interactions. The addition of tactile information during proprioceptive tasks provides a reduction in proprioceptive error: tactile cues reduces end-point error in matching tasks, continuous movement tasks, and point-to-point movement tasks (Lackner and Dizio, 1994; Tillery et al., 1994; Rao and Gordon, 2001). Even postural sway can be reduced by light tactile information that offers no mechanical support (Rabin et al., 2008). This multisensory integration is reinforced across the two-dimensional horizontal reaching space, as providing a tactile cue during a positional estimation task reduces error magnitude while maintaining the same spatial properties of the error (Rincon-Gonzalez et al., 2011). These studies suggest that proprioceptive estimation is a result of an internal reference of the body that incorporates internal tactile information. However, many of these prior results rely on a fingertip touching a tabletop, which could induce complex shear forces or small joint forces in addition to normal taction. The focus of this manuscript is to further investigate the effect of tactile input on multisensory integration: specifically we examined how proprioception would interact with artificial sensory substitution.

Sensory substitution has long been used as a method of improving or replacing lost sensory abilities. The implementation of vibrotactile or electrotactile stimulation has been used to replace vision, auditory, or other tactile deficiencies (Kaczmarek et al., 1991). Sensory substitution can also be used to associate actions to feedback, promoting action-sensation coupling and reinforcing body-ownership (Gapenne, 2014), which would promote effective feedback as long as artificial sensations do not disrupt any established proprioceptive estimation strategies. The effect of artificial tactile sensation on this multisensory integration is relatively unexplored. We have previously shown that hand posture can interfere with the perception of artificial sensation: a perceptual illusion elicited by sequential electrotactile stimulation of the fingertips can be abolished by assuming specific hand postures (Warren et al., 2010). Most of the literature investigates the efficiency of singular focused sensory substitution without considering the effects on multisensory integration.

By assessing the limitations and capabilities of vibrotactile and electrotactile sensory substitution, we can gain insight into this multisensory process. Thus, we aimed to investigate the effect artificial sensory substitution has on multisensory integration of tactile and proprioceptive information. We hypothesized that sensory substitution of vibrotactile and electrotactile information would not disrupt proprioceptive strategies and would integrate similarly to natural touch. We report that artificial tactile stimuli and proprioceptive estimation integrate appropriately, maintaining the non-uniform and idiosyncratic spatial distribution of endpoint errors, and reducing overall error although to a lesser degree than natural touch. We also report that sensory feedback increases estimation error in the non-dominant hand, but without changing the spatial properties of the endpoint error map.

## Materials and Methods

### Subjects

For this experiment, 22 subjects were recruited to perform a right-handed proprioceptive estimation task. Two subjects were excluded in the analysis due to handedness discrepancies, addressed below. The task, parameters, and experimental protocols were reviewed and approved by the Institutional Review Board at Arizona State University.

### Handedness

**FIGURE 1:**
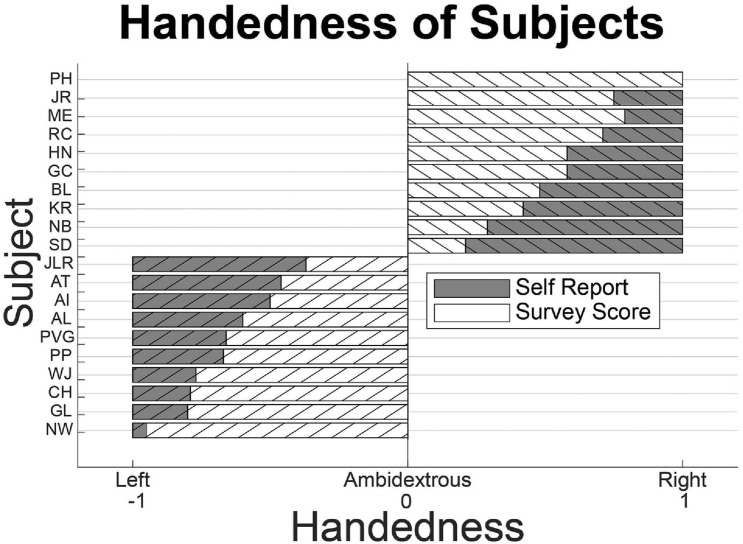
Subject handedness. Self-reporting and results from the Edinburgh Handedness Inventory questionnaire for included subjects. Only a single subject had questionnaire results that deviated from self-reporting. Another subject did not fulfill the questionnaire. Both were excluded from analysis.

Handedness was self-reported from each subject, and the Edinburgh Handedness Inventory questionnaire was used to follow-up and evaluate the handedness of each subject after the experiment (Oldfield, 1971; Figure 1). From subject self-reports, there were 11 right-handed subjects and 11 left-handed subjects. One self-reported right-handed subject failed to fill out the Edinburgh Handedness Inventory and that subject was excluded from the right-handed subjects. Only one subject that filled out the questionnaire had a result that differed from their self-report: a self-reported left-handed individual with a handedness score of 0.21. Due to this ambiguity, this subject was excluded from the left-handed subjects. This resulted in 20 subjects included in the analysis: 10 left-handed and 10 right-handed.

### Task

**FIGURE 2:**
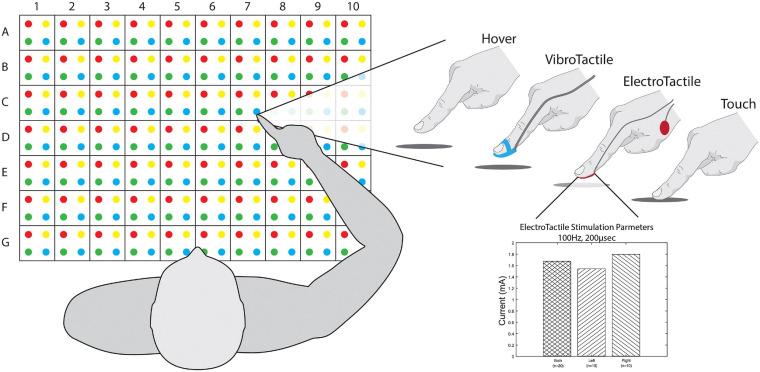
Workspace and feedback. **(Left)** Subjects sit in front of a grid of dots with 2.5 cm spacing between them, creating a workspace 50 cm wide and 35 cm deep. Targets and responses are referred to via their numerical value, alphabetical letter, and color, e.g., A1Red. **(Right)** Subject interaction for each feedback mode. Vibrotactile feedback required a custom 3D printed piece to hold the vibration motor to the fingertip with a Velcro strap. Electrotactile feedback electrode placement is indicated on the fingertip and ground on the head of the radius. **(Inset)** Electrotactile parameters for subjects. Tolerable current levels were chosen by each subject with a maximum of 2 mA.

Subjects sat in front of a table with a 50 cm wide and 35 cm deep grid, consisting of 280 targets with alphanumeric and color assignments on a 14 × 20 grid illustrated in Figure 2. A set of 75 random targets, with at least one from each alphanumeric square on the grid, were chosen for each subject and kept for all iterations of that subject’s experiment. For each trial of the task, the subject held their right hand a few centimeters above the edge of the table’s midline close to their chest. With the subject’s eyes closed, the experimenter guided the subject’s right hand to a randomly selected target, provided feedback for 5 s, and guided the hand back to the starting position. The subject then opened their eyes, and without moving their arm, reported the estimated target by alphanumeric value and color, e.g., “A1Red.” The overall procedure was explained to the subject, and each subject was administered at least one practice trial, depending on the subject’s self-reported confidence with respect to understanding the task. Trials were only aborted and repeated if the experimenter accidentally touched the subject’s hand to the table in the process of approaching the target.

The task was randomly delivered as four separate blocks for each feedback mode: hover, touch, electrotactile, and vibrotactile (Figure 2). Hover consisted of the subject keeping their hand above the table and receiving no tactile feedback before being guided back to the starting position. Touch consisted of the experimenter moving the subject’s hand to the target, then vertically lowering it to the table, allowing contact for 5 s and attempting to minimize any lateral or residual movement, vertically raising their hand, and then returning to the starting position. Vibrotactile feedback was delivered with a vibration motor in a 3D printed housing mounted to the finger with Velcro. Timing was controlled with an Arduino Uno microcontroller. Electrotactile stimulation was delivered via a Digitimer DS8R stimulator. Parameters of a biphasic, symmetrical pulse were set at 200 microsecond pulse width and subjective tolerable amplitudes. Pulse amplitude was determined for each subject, starting at 2 mA and lowered if needed until the subject reported the sensation as “tolerable but clear.” This provided a strong stimulus rather than a “just noticeable” stimulus. Each pulse with triggered via a TekTronix function generator at 100 Hz, generating 500 pulses over 5 s (Figure 2 Inset).

### Analysis

**FIGURE 3:**
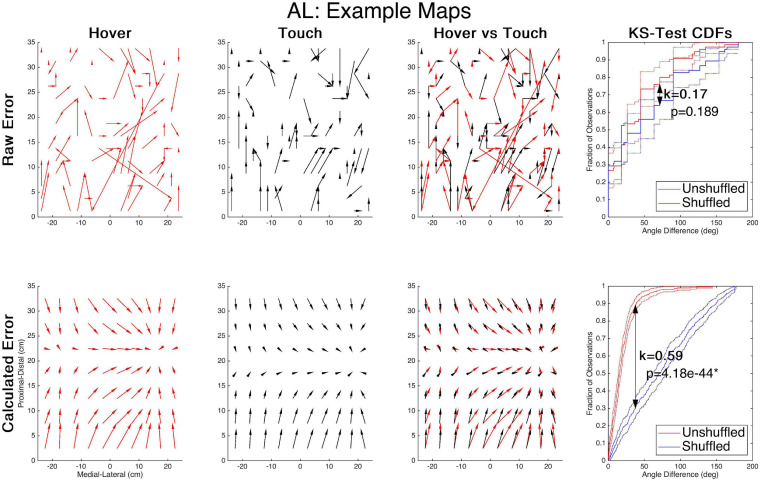
Data processing example. Arrows show proprioceptive error magnitude and direction for the hover condition. Each tail is the target and each head is the response. The top row shows the raw error of actual responses to targets for each condition and the comparison of the two. The bottom row shows the modeled error of the same conditions averaged for each alphanumeric grid. The far right column demonstrates the K-S test statistical process, relying on the maximum span between the cumulative distributions of differences in shuffled/unshuffled error angles, denoted as “k” and accompanied by the *p*-value of the test.

Using a subject’s estimations of the targets, the raw error magnitude and direction can be mapped across the sampled workspace. To obtain homogenous estimations across the entire workspace, the subjects’ responses to all 75 targets are used to create raw error vectors for each condition of each subject. The raw response vectors are separated into separate cartesian components: the lateral X component and distal-proximal Y component. The X and Y error are independently modeled using separate 4th order polynomial regressions and then X-error and Y-error are evaluated across each potential alphanumeric-color target. This provides a calculated error that exhibits a smoothing effect and diminishes representation of actual variability, but previous studies have not indicated a strong discrepancy of error magnitude or direction estimations between raw and calculated errors (Rincon-Gonzalez et al., 2011). We maintained the same target set across feedback modes to enable direct comparisons between these models. The use of this model provides the ability to measure evenly across the workspace and compare error vectors that are non-zero. Figure 3 illustrates this process for a single subject’s raw and calculated error from two feedback conditions. The first two columns illustrate the error maps alone, and the third column overlays the two maps for visual comparison. Both shape and magnitude need to be evaluated statistically, and all reported analyses were performed on each target’s calculated, generated from the polynomial models.

Data within subjects and across subjects were determined to have a non-normal distribution using the Lilliefors test, so non-parametric analyses were implemented. To determine if the shape of the error map was maintained between conditions, we employed the Kolmogorov-Smirnov (K-S) test which is described thoroughly in Rincon-Gonzalez et al. (2011). This test non-parametrically compares the distribution of two variables and determines if they are sampled from an identical distribution. In this application, we used it to compare the distribution of shuffled and unshuffled angular differences between error vectors; comparing actual angular differences to a random distribution. Significance would indicate the actual angular differences are significantly small and therefore imply a consistent shape. The unshuffled distribution represents each target’s error vectors’ actual angular difference between two feedback modes. A shuffled distribution was built by finding the angular difference between one feedback mode’s actual error vector and a randomly shuffled error vector for the second mode. If the unshuffled error vectors are similar, the angular difference is often small, creating a steep cumulative distribution function (CDF). Error maps with high variation in vector differences and a shuffled vector set would have CDFs that are more linear. Therefore, if the maximum difference between the CDFs of the shuffled and unshuffled sets is sufficiently large, then the error map shapes are significantly similar. An example of this is presented in the far-right column of Figure 3 for both raw and calculated error. This analysis works much better for the calculated error rather than the raw error, as the latter can contain error vectors of zero magnitude which prevent the accurate assessment of angular difference. To evaluate difference of error magnitude between pairs of feedback modes, Wilcoxon rank-sum tests were performed on the mean vector magnitudes between an individual’s estimations. To address the multiple comparisons between feedback conditions (6 total), we used α = 0.05/6 as a Bonferroni correction for both Wilcoxon rank-sum tests and the K-S tests.

To explore condition effects more thoroughly, Friedman tests were implemented on feedback modes within handedness and handedness within feedback modes.

## Results

To evaluate the effect of sensory substitution on the map of proprioceptive error, the magnitude and shape of error were tested between feedback conditions. These experiments provided raw error across 75 targets of the workspace (Figure 3, Top Row). The tests were then performed on 4th order polynomial models of each subjects’ X and Y error, evaluated at each of the 280 target locations (Figure 3, Bottom Row and simplified to just the alphanumeric grid). R^2^ coefficients of the 4th order polynomial fits were at minimum 0.86, averaged 0.96, and are displayed in Table 1. Using the resultant vectors, a Kolmogorov-Smirnov test compared the spatial structure between feedback conditions and a Wilcoxon rank-sum compared the magnitude of error between feedback conditions.

**TABLE 1:** Regression coefficients for polynomial fits.

	**Subjects**		**Hover**	**Touch**	**Electro**	**Vibro**		**Subjects**		**Hover**	**Touch**	**Electro**	**Vibro**
Right-handed	BL	X	0.97	0.97	0.95	0.96	Left-handed	JLR	X	0.96	0.94	0.94	0.97
		Y	0.94	0.95	0.94	0.96			Y	0.96	0.95	0.96	0.94
	GC	X	0.96	0.97	0.98	0.89		NW	X	0.97	0.99	0.98	0.98
		Y	0.98	0.97	0.97	0.96			Y	0.97	0.97	0.99	0.97
	HN	X	0.97	0.96	0.96	0.96		PKP	X	0.96	0.98	0.98	0.98
		Y	0.94	0.97	0.97	0.96			Y	0.96	0.97	0.98	0.97
	JR	X	0.94	0.94	0.94	0.96		WJ	X	0.97	0.97	0.98	0.97
		Y	0.97	0.94	0.95	0.96			Y	0.94	0.98	0.98	0.97
	KR	X	0.95	0.96	0.96	0.95		GL	X	0.97	0.96	0.95	0.97
		Y	0.97	0.94	0.97	0.98			Y	0.93	0.96	0.97	0.98
	ME	X	0.95	0.97	0.97	0.98		AI	X	0.97	0.95	0.86	0.91
		Y	0.96	0.98	0.97	0.97			Y	0.97	0.96	0.94	0.94
	NB	X	0.97	0.93	0.95	0.95		AL	X	0.91	0.98	0.97	0.94
		Y	0.97	0.96	0.91	0.96			Y	0.93	0.97	0.92	0.97
	PH	X	0.9	0.96	0.96	0.96		AT	X	0.86	0.96	0.96	0.96
		Y	0.95	0.95	0.97	0.94			Y	0.88	0.96	0.97	0.97
	RC	X	0.98	0.99	0.99	0.99		CH	X	0.96	0.97	0.95	0.97
		Y	0.96	0.99	0.99	0.98			Y	0.97	0.97	0.93	0.97
	SRD	X	0.96	0.98	0.98	0.95		PVG	X	0.96	0.97	0.98	0.96
		Y	0.95	0.96	0.96	0.98			Y	0.96	0.97	0.96	0.98

*Modeled error for X and Y components of raw error vectors (target to response) for each subject in each condition.*

**FIGURE 4:**
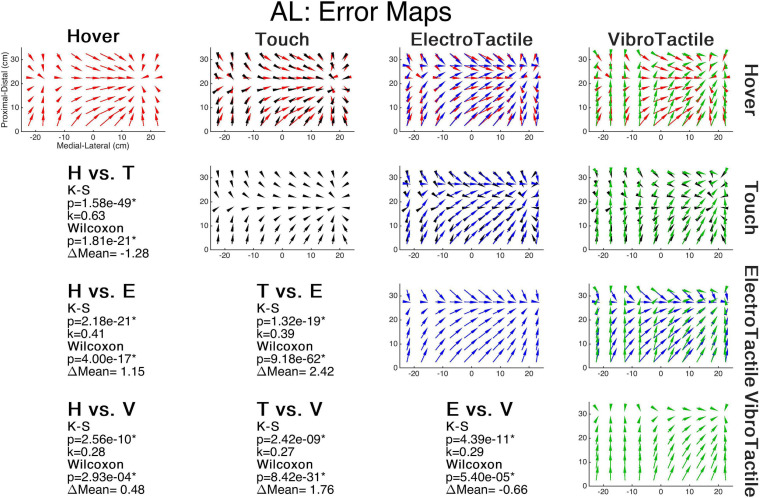
Error maps comparisons. (Diagonal) Error maps of proprioceptive error direction for individual feedback conditions across the workspace for a single subject. (Upper Triangular) Each error map comparison between two feedback modes. (Lower Triangular) Each comparison’s statistical results. K-S significance implies the maps possess statistically similar shapes. Wilcoxon rank-sum significance implies a difference in the error magnitudes, where positive ΔMean values indicate increased error in the latter mode.

Figure 4 illustrates the error maps of each mode and the comparisons between each condition for a single subject, as well as that subject’s statistical results. The visualization suggests that the spatial structure is similar across all modes and the significant K-S test results corroborate. Table 2 outlines the statistical results of both tests with respective *k* values and change in mean error (ΔM). Comparing vector directions between feedback conditions (6 comparisons) for each subject (20 subjects) using the K-S test showed significantly similar spatial structures in 119/120 cases, with only one hover and vibrotactile comparison producing insignificant results. This case was a left-handed subject performing the task. Overall, sensory substitution does not appear to disrupt the spatial structure of proprioceptive error in this task.

**TABLE 2:** Statistical results between conditions.

	**Subjects**	**Hover vs. Touch**	**Hover vs. Electro**	**Hover vs. Vibro**	**Touch vs. Electro**	**Touch vs. Vibro**	**Electro vs. Vibro**
Right-handed	BL	ΔM = 1.76 cm (*p* < 0.05/6)	ΔM = 0.51 cm (*p* < 0.05/6)	ΔM = 0.99 cm (*p* < 0.05/6)	ΔM = −1.25 cm (*p* < 0.05/6)	ΔM = −0.77 cm (*p* < 0.05/6)	ΔM = 0.48 cm (*p* < 0.05/6)
		*k* = 0.63 (*p* < 0.05/6)	*k* = 0.62 (*p* < 0.05/6)	*k* = 0.47 (*p* < 0.05/6)	*k* = 0.73 (*p* < 0.05/6)	*k* = 0.44 (*p* < 0.05/6)	*k* = 0.39 (*p* < 0.05/6)
	GC	ΔM = −1.6 cm (*p* < 0.05/6)	ΔM = 0.19 cm (*p* = 0.33)	ΔM = −0.45 cm (*p* < 0.05/6)	ΔM = 1.79 cm (*p* < 0.05/6)	ΔM = 1.14 cm (*p* < 0.05/6)	ΔM = −0.64 cm (*p* < 0.05/6)
		*k* = 0.33 (*p* < 0.05/6)	*k* = 0.23 (*p* < 0.05/6)	*k* = 0.34 (*p* < 0.05/6)	*k* = 0.37 (*p* < 0.05/6)	*k* = 0.42 (*p* < 0.05/6)	*k* = 0.34 (*p* < 0.05/6)
	HN	ΔM = −0.69 cm (*p* < 0.05/6)	ΔM = −1.81 cm (*p* < 0.05/6)	ΔM = −0.3 cm (*p* = 0.12)	ΔM = −1.12 cm (*p* < 0.05/6)	ΔM = 0.39 cm (*p* = 0.15)	ΔM = 1.51 cm (*p* < 0.05/6)
		*k* = 0.53 (*p* < 0.05/6)	*k* = 0.48 (*p* < 0.05/6)	*k* = 0.51 (*p* < 0.05/6)	*k* = 0.62 (*p* < 0.05/6)	*k* = 0.53 (*p* < 0.05/6)	*k* = 0.48 (*p* < 0.05/6)
	JR	ΔM = −0.43 cm (*p* < 0.05/6)	ΔM = 1.25 cm (*p* < 0.05/6)	ΔM = 0.1 cm (*p* = 0.7)	ΔM = 1.69 cm (*p* < 0.05/6)	ΔM = 0.53 cm (*p* < 0.05/6)	ΔM = −1.16 cm (*p* < 0.05/6)
		*k* = 0.2 (*p* < 0.05/6)	*k* = 0.2 (*p* < 0.05/6)	*k* = 0.17 (*p* < 0.05/6)	*k* = 0.25 (*p* < 0.05/6)	*k* = 0.55 (*p* < 0.05/6)	*k* = 0.29 (*p* < 0.05/6)
	KR	ΔM = −0.92 cm (*p* < 0.05/6)	ΔM = −1.64 cm (*p* < 0.05/6)	ΔM = −1.14 cm (*p* < 0.05/6)	ΔM = −0.72 cm (*p* < 0.05/6)	ΔM = −0.22 cm (*p* = 0.02)	ΔM = 0.5 cm (*p* < 0.05/6)
		*k* = 0.49 (*p* < 0.05/6)	*k* = 0.61 (*p* < 0.05/6)	*k* = 0.62 (*p* < 0.05/6)	*k* = 0.56 (*p* < 0.05/6)	*k* = 0.57 (*p* < 0.05/6)	*k* = 0.65 (*p* < 0.05/6)
	ME	ΔM = −1.67 cm (*p* < 0.05/6)	ΔM = 0.21 cm (*p* < 0.05/6)	ΔM = −0.75 cm (*p* < 0.05/6)	ΔM = 1.88 cm (*p* < 0.05/6)	ΔM = 0.92 cm (*p* < 0.05/6)	ΔM = −0.96 cm (*p* < 0.05/6)
		*k* = 0.5 (*p* < 0.05/6)	*k* = 0.45 (*p* < 0.05/6)	*k* = 0.34 (*p* < 0.05/6)	*k* = 0.33 (*p* < 0.05/6)	*k* = 0.34 (*p* < 0.05/6)	*k* = 0.38 (*p* < 0.05/6)
	NB	ΔM = 1.2 cm (*p* < 0.05/6)	ΔM = 1.16 cm (*p* < 0.05/6)	ΔM = 0.46 cm (*p* = 0.18)	ΔM = −0.04 cm (*p* = 0.73)	ΔM = −0.74 cm (*p* < 0.05/6)	ΔM = −0.7 cm (*p* < 0.05/6)
		*k* = 0.5 (*p* < 0.05/6)	*k* = 0.36 (*p* < 0.05/6)	*k* = 0.47 (*p* < 0.05/6)	*k* = 0.46 (*p* < 0.05/6)	*k* = 0.68 (*p* < 0.05/6)	*k* = 0.55 (*p* < 0.05/6)
	PH	ΔM = −3.22 cm (*p* < 0.05/6)	ΔM = −1.66 cm (*p* < 0.05/6)	ΔM = −1.59 cm (*p* < 0.05/6)	ΔM = 1.56 cm (*p* < 0.05/6)	ΔM = 1.62 cm (*p* < 0.05/6)	ΔM = 0.07 cm (*p* = 0.8)
		*k* = 0.47 (*p* < 0.05/6)	*k* = 0.66 (*p* < 0.05/6)	*k* = 0.58 (*p* < 0.05/6)	*k* = 0.43 (*p* < 0.05/6)	*k* = 0.37 (*p* < 0.05/6)	*k* = 0.77 (*p* < 0.05/6)
	RC	ΔM = −1.36 cm (*p* < 0.05/6)	ΔM = −1.21 cm (*p* < 0.05/6)	ΔM = −0.37 cm (*p* < 0.05/6)	ΔM = 0.15 cm (*p* < 0.05/6)	ΔM = 0.99 cm (*p* < 0.05/6)	ΔM = 0.84 cm (*p* < 0.05/6)
		*k* = 0.24 (*p* < 0.05/6)	*k* = 0.3 (*p* < 0.05/6)	*k* = 0.43 (*p* < 0.05/6)	*k* = 0.16 (*p* < 0.05/6)	*k* = 0.27 (*p* < 0.05/6)	*k* = 0.34 (*p* < 0.05/6)
	SRD	ΔM = 0.39 cm (*p* < 0.05/6)	ΔM = −0.2 cm (*p* < 0.05/6)	ΔM = 0.26 cm (*p* = 0.04)	ΔM = −0.59 cm (*p* < 0.05/6)	ΔM = −0.13 cm (*p* < 0.05/6)	ΔM = 0.46 cm (*p* < 0.05/6)
		*k* = 0.33 (*p* < 0.05/6)	*k* = 0.28 (*p* < 0.05/6)	*k* = 0.23 (*p* < 0.05/6)	*k* = 0.22 (*p* < 0.05/6)	*k* = 0.19 (*p* < 0.05/6)	*k* = 0.26 (*p* < 0.05/6)
Left-handed	JLR	ΔM = 0.23 cm (*p* = 0.04)	ΔM = −1.04 cm (*p* < 0.05/6)	ΔM = −1.06 cm (*p* < 0.05/6)	ΔM = −1.27 cm (*p* < 0.05/6)	ΔM = −1.28 cm (*p* < 0.05/6)	ΔM = −0.02 cm (*p* = 0.9)
		*k* = 0.64 (*p* < 0.05/6)	*k* = 0.46 (*p* < 0.05/6)	*k* = 0.39 (*p* < 0.05/6)	*k* = 0.62 (*p* < 0.05/6)	*k* = 0.5 (*p* < 0.05/6)	*k* = 0.56 (*p* < 0.05/6)
	NW	ΔM = 0.07 cm (*p* = 0.01)	ΔM = −0.54 cm (*p* < 0.05/6)	ΔM = 0.13 cm (*p* = 0.14)	ΔM = −0.61 cm (*p* < 0.05/6)	ΔM = 0.07 cm (*p* = 0.41)	ΔM = 0.68 cm (*p* < 0.05/6)
		*k* = 0.38 (*p* < 0.05/6)	*k* = 0.4 (*p* < 0.05/6)	*k* = 0.36 (*p* < 0.05/6)	*k* = 0.6 (*p* < 0.05/6)	*k* = 0.77 (*p* < 0.05/6)	*k* = 0.5 (*p* < 0.05/6)
	PKP	ΔM = −0.3 cm (*p* < 0.05/6)	ΔM = −0.86 cm (*p* < 0.05/6)	ΔM = −1.07 cm (*p* < 0.05/6)	ΔM = −0.55 cm (*p* < 0.05/6)	ΔM = −0.77 cm (*p* < 0.05/6)	ΔM = −0.21 cm (*p* = 0.05)
		*k* = 0.36 (*p* < 0.05/6)	*k* = 0.49 (*p* < 0.05/6)	*k* = 0.42 (*p* < 0.05/6)	*k* = 0.42 (*p* < 0.05/6)	*k* = 0.45 (*p* < 0.05/6)	*k* = 0.47 (*p* < 0.05/6)
	WJ	ΔM = 1.25 cm (*p* < 0.05/6)	ΔM = 0.14 cm (*p* = 0.06)	ΔM = 1.35 cm (*p* < 0.05/6)	ΔM = −1.12 cm (*p* < 0.05/6)	ΔM = 0.1 cm (*p* = 0.53)	ΔM = 1.22 cm (*p* < 0.05/6)
		*k* = 0.33 (*p* < 0.05/6)	*k* = 0.33 (*p* < 0.05/6)	*k* = 0.38 (*p* < 0.05/6)	*k* = 0.44 (*p* < 0.05/6)	*k* = 0.64 (*p* < 0.05/6)	*k* = 0.43 (*p* < 0.05/6)
	GL	ΔM = 1.35 cm (*p* < 0.05/6)	ΔM = 2.02 cm (*p* < 0.05/6)	ΔM = 2.68 cm (*p* < 0.05/6)	ΔM = 0.67 cm (*p* < 0.05/6)	ΔM = 1.32 cm (*p* < 0.05/6)	ΔM = 0.66 cm (*p* = 0.02)
		*k* = 0.58 (*p* < 0.05/6)	*k* = 0.56 (*p* < 0.05/6)	*k* = 0.41 (*p* < 0.05/6)	*k* = 0.68 (*p* < 0.05/6)	*k* = 0.49 (*p* < 0.05/6)	*k* = 0.52 (*p* < 0.05/6)
	AI	ΔM = 1.71 cm (*p* < 0.05/6)	ΔM = 0.56 cm (*p* < 0.05/6)	ΔM = 0.71 cm (*p* < 0.05/6)	ΔM = −1.16 cm (*p* < 0.05/6)	ΔM = −1.01 cm (*p* < 0.05/6)	ΔM = 0.15 cm (*p* = 0.01)
		*k* = 0.35 (*p* < 0.05/6)	*k* = 0.3 (*p* < 0.05/6)	*k* = 0.39 (*p* < 0.05/6)	*k* = 0.34 (*p* < 0.05/6)	*k* = 0.28 (*p* < 0.05/6)	*k* = 0.47 (*p* < 0.05/6)
	AL	ΔM = −1.28 cm (*p* < 0.05/6)	ΔM = 1.15 cm (*p* < 0.05/6)	ΔM = 0.48 cm (*p* < 0.05/6)	ΔM = 2.42 cm (*p* < 0.05/6)	ΔM = 1.76 cm (*p* < 0.05/6)	ΔM = −0.66 cm (*p* < 0.05/6)
		*k* = 0.63 (*p* < 0.05/6)	*k* = 0.42 (*p* < 0.05/6)	*k* = 0.57 (*p* < 0.05/6)	*k* = 0.31 (*p* < 0.05/6)	*k* = 0.5 (*p* < 0.05/6)	*k* = 0.43 (*p* < 0.05/6)
	AT	ΔM = −0.07 cm (*p* = 0.11)	ΔM = 1.18 cm (*p* < 0.05/6)	ΔM = −0.54 cm (*p* < 0.05/6)	ΔM = 1.26 cm (*p* < 0.05/6)	ΔM = −0.47 cm (*p* < 0.05/6)	ΔM = −1.72 cm (*p* < 0.05/6)
		*k* = 0.23 (*p* < 0.05/6)	*k* = 0.12 (*p* = 0.04)	*k* = 0.09 (*p* = 0.17)	*k* = 0.2 (*p* < 0.05/6)	*k* = 0.36 (*p* < 0.05/6)	*k* = 0.18 (*p* < 0.05/6)
	CH	ΔM = −1.33 cm (*p* < 0.05/6)	ΔM = 0.39 cm (*p* = 0.07)	ΔM = 0.16 cm (*p* = 0.29)	ΔM = 1.73 cm (*p* < 0.05/6)	ΔM = 1.49 cm (*p* < 0.05/6)	ΔM = −0.23 cm (*p* = 0.74)
		*k* = 0.5 (*p* < 0.05/6)	*k* = 0.25 (*p* < 0.05/6)	*k* = 0.4 (*p* < 0.05/6)	*k* = 0.3 (*p* < 0.05/6)	*k* = 0.42 (*p* < 0.05/6)	*k* = 0.41 (*p* < 0.05/6)
	PVG	ΔM = 0.54 cm (*p* < 0.05/6)	ΔM = 0.61 cm (*p* < 0.05/6)	ΔM = 1.01 cm (*p* < 0.05/6)	ΔM = 0.06 cm (*p* = 0.52)	ΔM = 0.46 cm (*p* = 0.01)	ΔM = 0.4 cm (*p* = 0.01)
		*k* = 0.63 (*p* < 0.05/6)	*k* = 0.42 (*p* < 0.05/6)	*k* = 0.57 (*p* < 0.05/6)	*k* = 0.31 (*p* < 0.05/6)	*k* = 0.5 (*p* < 0.05/6)	*k* = 0.43 (*p* < 0.05/6)

*Difference in means and Wilcoxon rank-sum tests significance in parentheses on top. K-S test k value and significance on bottom. A negative ΔM value implies the latter has decreased error. Any non-significant comparisons are indicated in gray. Used alpha of 0.05/6 for multiple comparison corrections.*

Comparing error magnitude between feedback modes for each subject produces significant results in 96/120 tests. Specifically, Rincon-Gonzalez et al. showed that the touch condition had lower mean error than hover. We observed here a significant decrease in error in 7 out of 10 right-handed subjects, and 3 out of 7 left-handed subjects. We also carried out a Friedman test and a Kruskal-Wallis test, respectively, with feedback mode and handedness as primary factors. Both produced significant results: the test of differences between handedness rendered a Chi-square value of 109.75 (*p* << 0.05) and the test of differences between feedback mode rendered a Chi-square value of 117.75 (*p* << 0.05). Dunn-Sidak *post-hoc* tests produced no significant results in the feedback comparisons. To investigate further Friedman tests were performed across feedback mode within handedness, and Wilcoxan Ranksum tests across handedness within each feedback mode. For left-handed subjects, a test of differences between feedback mode rendered a Chi-square value of 78.19 (*p* << 0.05). For right-handed subjects, a test of differences between feedback mode rendered a Chi-square value of 268.84 (*p* << 0.05). Respectively, for the hover, electrotactile, vibrotactile, and touch conditions, respective Wilcoxon rank-sum test of differences between handedness rendered *Z* = 3.08 (*p* << 0.05), *Z* = -6.46 (*p* << 0.05), *Z* = 5.44 (*p* << 0.05), and *Z* = -12.14 (*p* << 0.05).

**FIGURE 5:**
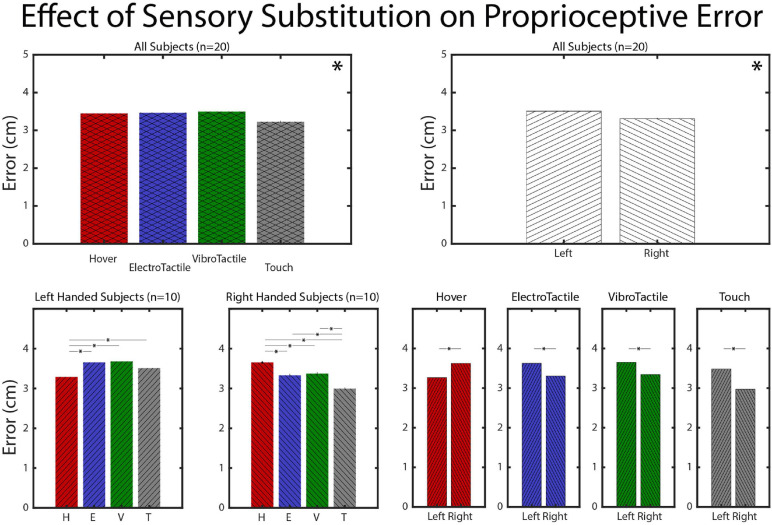
Mean error comparisons. Proprioceptive estimation error separated by handedness and averaged across all subjects or subjects by handedness (* indicates significance for the appropriate test).

Trends of mean error and indications of *post-hoc* Dunn-Sidak tests’ significance are provided in Figure 5. For the hover feedback mode, left-handed subjects demonstrate significantly less error than right-handed subjects while all other sensory feedback modes showed significantly more error for left-handed subjects. Also, in left-handed subjects, all sensory feedback modes produced higher mean error than the hover condition. Right-handed subjects produced opposing results: all feedback modes produced less mean error than the hover condition with the touch condition producing significantly less than the sensory substation modes. Thus, sensory substitution slightly improved error for right-handed subjects performing with their dominant hand, but not as much as actual touch. It also suggests that left-handed subjects performing a spatial task with their non-dominant hand are more accurate than right-handed subjects performing with their dominant hand with no feedback. Sensory feedback appears to disrupt non-dominant hand location estimation strategies while bolstering dominant hand strategies.

## Discussion

For 99% of the comparisons of the error map shape, the introduction of artificial or natural tactile information did not provide a disruption, which indicates the synergistic multisensory integration of taction and proprioception and therefore supports the viable use of sensory substitution in terms of limited degradation to proprioceptive processing. Error maps were idiosyncratic and stable across conditions, confirming previous literature (Rincon-Gonzalez et al., 2011, 2012). This demonstrates that any multisensory integration that does occur does not alter the already established underlying mapping between sensory inputs and hand.

For error magnitude, handedness played an interesting role in the results. The task was completed with the right hand, so right-handed subjects performed it with their dominant hand and left-handed subjects performed with their non-dominant hand. Only the right-handed subjects demonstrated a significant reduction in error from the Hover condition to the Touch condition (Figure 5, bottom second from left). Left-handed subjects did not exhibit this improvement. However, left-handed subjects performed with less overall error than right-handed subjects (Figure 5, top right). This could be consonant with the hypothesis that the dominant hemisphere/limb system is used for trajectory/velocity control and the non-dominant system is used for positional control (Sainburg, 2005). Our experiment is fundamentally positional and it follows that the subjects performing with their non-dominant limb system would demonstrate better positional estimation.

Both vibrotactile and electrotactile sensory substitution modes result in error magnitudes that are statistically different from each other across handedness. For the right-handed subjects, the sensory substitution modes produce error that is significantly lower than the Hover condition and significantly higher than the Touch condition, evidencing multisensory integration but of lower quality than natural touch. However, the left-handed subject’s positional error is increased during any sensory feedback condition. In this result, we observe antagonistic multisensory integration hindering the non-dominant positional estimation ability. As non-dominant limb system prioritize positional control, vibrational, or deterministic stimulation patterns may activate proprioceptive receptors via tissue transduction and confound the system in a way for which the dominant limb system can account. In this vein, successfully multisensory integration of taction and proprioception appears to rely on the interaction of task context (positional vs. velocity) and taction mode (natural vs. artificial). Investigation into this relationship with Semmes-Weinstein filaments is immediately prudent, to utilize tactile stimulation that can be perceived with minimal joint or proprioceptive activation.

While error maps are stable across all investigated feedback modalities, sensory substitution fails to achieve the same multisensory integration as normal touch: error is not reduced to the same level for dominant hand cases and error is increased for non-dominant hand cases. The overall limitation is likely due to familiarity with the stimulation percept and lack of training to use it practically. The handedness discrepancy is likely from existing properties of the proprioceptive system. We know that tactile sensory substitution can help with proprioceptive movements (Bark et al., 2015) but it fails to replace proprioceptive positional estimation (Hasson and Manczurowsky, 2015). As the non-dominant hand prioritizes positional control, the unfamiliarity of the sensation appears to have a more profound and deleterious effect on the subjects’ ability to estimate position. Finally, our results show that the effect of tactile stimulation on proprioceptive mapping is not simply dependent on unstructured tactile information from the fingertip: actual contact with a stable substrate seems to provide the best cue to enhancing proprioceptive estimation.

## Conclusion

In this study, we examined the role of multiple feedback modalities on multisensory integration of tactile and proprioceptive information. By reconstructing the end-point estimation error across a two-dimensional workspace, we were able to statistically compare error magnitude and error map shape. We were also able to investigate the role handedness had on the multisensory integration of the multiple feedback modes, which provided an unexpected insight into proprioceptive prioritization. Vibrotactile and electrotactile sensory substitution does not disrupt estimation strategies across the workspace. For the dominant hand, this feedback does provide significant improvements in positional estimation error, but not to levels seen with multisensory integration of normal touch. Non-dominant hand positional error was significantly exacerbated even though normal touch beneficially integrates.

Successful multisensory integration of touch and proprioception appears to be a function of taction mode. It is unknown if the limitation is perceptual due to non-familiar or non-practical sensations, neurophysiological due to excessive mechanoreceptor/nerve recruitment from mechanical or electrical transduction, or if normal multisensory integration requires the utilization of arm and digit joint forces. These results also suggests that sensory substitution may hinder positional ability in practical application, such as prosthetics, during non-dominant tasks even if it does not hinder overall estimation strategy. If prostheses or other haptic environments integrate sensory feedback, it is imperative that such feedback not produce a worse result than the absence of any feedback. This should be addressed in the design, assignment, and training of feedback so that the users are aware of the limitation.

## Data Availability Statement

The raw data supporting the conclusions of this article will be made available by the authors, without undue reservation.

## Ethics Statement

The studies involving human participants were reviewed and approved by the Arizona State University Institutional Review Board. The patients/participants provided their written informed consent to participate in this study.

## Author Contributions

All authors contributed to experimental design, experimental execution, data analysis, and drafting the manuscript.

## Conflict of Interest

The authors declare that the research was conducted in the absence of any commercial or financial relationships that could be construed as a potential conflict of interest.
